# Cervical Cancer Staging in Saudi Arabia Clinicoradiological Correlation

**DOI:** 10.1155/2019/8745828

**Published:** 2019-06-23

**Authors:** Nisreen Anfinan

**Affiliations:** Gynecology Oncology Unit, Department of Obstetrics and Gynaecology, Faculty of Medicine, King Abdulaziz University, Jeddah, Saudi Arabia

## Abstract

**Objective:**

The aim of this study was to compare the finding of pelvic MRI with clinical staging using cystoscopy and sigmoidoscopy for cervical cancer patients.

**Method:**

We reviewed all patients with cervical cancer between January 2001 and December 2015. We correlate the clinical examination, cystoscopy, and sigmoidoscopy with MRI findings.

**Result:**

A total of 152 patients were enrolled. 114 (74.9%) were with locally advanced cervix cancer. The true positives for MRI in the detection of parametrium were in 94 patients, with sensitivity, specificity, positive, PPV, and NPV of 72%, 82%, 96%, and 33%, respectively. The false negative of the MRI to detect the bladder invasion was 2. Nineteen patients reported having bladder invasion on MRI not confirmed by cystoscopy. None of the patients who had a negative rectal invasion by MRI were found to have rectal involvement by sigmoidoscopy with a specificity of 91%.

**Conclusion:**

The combined MRI and clinical staging for parametrial evaluation should still be carried out for the staging of cervical cancer. However, in the absence of the bladder and the rectal invasion in the MRI, it will be safe to avoid the need for a cystoscopy and/or sigmoidoscopy for complete staging in the majority of patients with cervical cancer.

## 1. Introduction

The precise tumor staging in patients with cervical cancer is crucial for an appropriate therapy. Traditionally, cervical cancer staging is performed with a pelvic clinical examination that includes inspection and palpation (if necessary under anesthesia) with special consideration to tumor size, vaginal involvement, and parametrial and pelvic sidewall status, as well as cystoscopy, sigmoidoscopy, intravenous urography (IVU), and radiological evaluation of the lung [1]. Staging of cervical cancer is executed in compliance with the guidelines established by the International Federation of Gynecology and Obstetrics (FIGO) reviewed in 2009 [2]. In case of tumor being clinically limited to the cervix (≤ stage IIA), the preferred treatment is surgery in most patients. When the tumor extends clinically to the parametrium (late stage ≥ stage IIB), primary chemoradiation is the treatment of choice [1, 3, 4]. However, clinical staging is subject to inaccuracy with high error rates ranging between 26 and 66% [5–10].

It has been shown that magnetic resonance imaging (MRI) is a reliable investigation to assess the tumor invasiveness and may provide accurate staging for cervical cancer [3, 9, 11–13].

We carried out this study to present our experience with the use of MRI in the staging of patients with cervical cancer with reference to the standard clinical examination with respect to local pelvic involvement, as well as bladder and rectal invasion.

## 2. Material and Methods

We conducted a retrospective review of all histologically confirmed cases of cervical cancer who were treated at King Abdulaziz University Hospital, Jeddah, Saudi Arabia, in the period from January 2001 to December 2015. There were 152 patients who completed their final stage through the standard clinical examination and the MRI pelvis. Data were collected, including age, parity, and the clinical and MRI findings. The staging was carried out according to the FIGO staging system [2].

We correlated the MRI finding with respect to the vagina, parametrium, and pelvic side wall involvement as well as bladder and rectal involvement of 152 patients with the standard clinical examination under anesthesia, cystoscopy, and sigmoidoscopy.

Sensitivity, specificity, and negative (NPV) and positive (PPV) predictive values were computed using the SPSS software program.

## 3. Result

The population's median age was 53 years (range: 20-90 years). The median parity was four with a range between 0 and 14. The median BMI was 26.6 (range 14.8-45.4). Of the total patients, 17 (11.2%) were stage IB and 114 (74.9%) were at locally advanced stage of cervix cancer. Bladder involvement was found among 11 (7.2%) patients and distal metastasis was found in 10 (6.6%) (Table 1).

The most common histopathological type was squamous cell carcinoma in 128 (84.2%), followed by adenocarcinoma in 20 (13.4%) of the patients. Two patients were with adenosquamous carcinoma and one was with leiomyosarcoma and non-Hodgkin's lymphoma was in the other patient (Table 2).

The correlation between MRI and the standard clinical examination is depicted in Table 3.

The vagina was involved in 17 cases, as identified by clinical examination but not observed in MRI with the sensitivity of 67% and the specificity of 60%. The number of true positives of MRI findings in the detection of parametrium was 94, resulting in sensitivity 72%, specificity 82%, PPV 96%, and NPV 33%.

The specificity of MRI to detect pelvic sidewall disease was 94% with PPV 77%. Regarding detection of bladder invasion, MRI yielded 2 false negatives and 19 patients, who were positively detected by MRI, were not confirmed by cystoscopy, with sensitivity of 78%, specificity 87%, and NPV 98%.

None of the patients who had a negative rectal invasion by MRI was found to have rectal involvement by sigmoidoscopy with a specificity of 91%.

## 4. Discussion

There are numerous prognostic factors that predict the outcome of cervical cancer and can affect treatment planning. Tumor histopathology is an important prognostic factor; and squamous cell carcinoma represents the most frequent histopathological type in this study. Other reports revealed similar incidence rates between 80 and 90% [14]. The tumor stage constitutes an additional prognostic factor [15]. Similar to what has been reported in Saudi Arabia by Manji in 1998 [16], our study showed that 88.8% were diagnosed at an advanced stage using FIGO clinical staging; this was due to lack of cervical screening program in Saudi Arabia.

Parametrial invasion is considered a determining factor for appropriate treatment choice, commonly surgery or radiotherapy. MRI diagnostic performance showed high NPV of 94-100%, indicating its high reliability in excluding parametrial invasion [7, 17]. Other studies reported that MRI has 69% and 93% sensitivity and specificity, respectively, to detect parametrial invasion [18, 19]. In our study population, MRI had a sensitivity of 72%, but specificity was 82%. Only four false positive findings occurred with MRI, while it failed to detect parametrial involvement in 36 patients resulting in very low NPV of 33%.

MRI performance in detecting vaginal invasion is reported to be adequate, with a general accuracy of 86%-93% [20, 21]. We observed a slight decrease in accuracy with a sensitivity of 67% and a positive predictive value of 47%.

The reported sensitivity and specificity of MRI in detecting the bladder and rectum invasion are 71%-100% and 88%-91%, respectively [22, 23]. Bladder and rectal involvement can be confidently excluded in MRI with 100% NPV, making cystoscopy and sigmoidoscopy redundant [22]. In our study, the bladder NPV and rectal invasion are 98%-100%, respectively, which support the previous finding. We also found that, in our experience, the MRI did not detect bladder invasion in two patients and 19 patients who reported having bladder invasion on MRI were not confirmed by cystoscopy, which means that the MRI can replace the cystoscopic examination for the evaluation of the bladder. However, this must be determined by further study.

In conclusion, the majority of cervical cancer patients in our study population were diagnosed at an advanced stage. There was discrepancy between the MRI finding and the clinical examination for the evaluation of vaginal and parametrial involvement, and that is why combined MRI and clinical staging are still required for adequate evaluation of parametrial and vaginal invasion. In absence of MRI evidence of bladder and rectal invasion, performing cystoscopy and/or sigmoidoscopy for complete staging can be safely waived in majority of cervical cancer patients.

*Limitations of the Study*. Our study involved small number of patients and the correlation was limited, since we did not have histopathological confirmation of the parametrium and vagina invasion, which is not required by FIGO staging.

## Figures and Tables

**Table 1:** Cervical cancer Stage (Total. 152).

	No	%
IB	17	11.2
IIA	4	2.6
IIB	80	52.6
IIIB	30	19.7
IVA	11	7.2
IV B	10	6.6

**Table 2:** Histopathology of Cervical Cancer (Total 152).

Histopathology	No.	%
Squamous cell Carcinoma	128	84.2%
Adeno Carcinoma	20	13.2%
Adenosquamous carcinoma	2	1.3%
Others	2	1.3%

**Table 3:** Correlation between Clinical Staging and MRI finding in cervical cancer patients.

MR imaging	A number of:				Sensitivity (95% CI)	Specificity (95% CI)	Positive predictive value (95% CI)	Negative predictive value (95% CI)
	True positives	False positives	False Negatives	True Negatives				
Parametrial	94	4	36	18	0.72 (0.65 – 0.8)	0.82 (0.66 – 0.96)	0.96 (0.92-1)	0.33 (0.21-0.46)
Pelvic Side Wall	23	7	18	104	0.56 (0.41 – 0.71)	0.94 (0.89 – 0.98)	0.77 (0.62-0.92)	0.85 (0.79-0.92)
Bladder invasion	7	19	2	122	0.78 (0.5 – 1)	0.87 (0.8 – 0.92)	0.27 (0.1-0.44)	0.98 (0.96-1)
Rectal invasion	1	8	0	140	0.75 (0.13 – 0.15)	0.91 (0.98 – 1)	0.15 (00-0.37)	0.99 (0.99-1)
Vaginal involvement	34	38	17	56	0.67 (0.54-0.8)	0.6 (0.5-0.7)	0.47 (0.4-0.6)	0.77 (0.67-0.86)

## Data Availability

The data used to support the findings of this study are available from the corresponding author upon request.
